# Acceleration Gait Measures as Proxies for Motor Skill of Walking: A Narrative Review

**DOI:** 10.1109/TNSRE.2020.3044260

**Published:** 2021-03-01

**Authors:** Pritika Dasgupta, Jessie VanSwearingen, Alan Godfrey, Mark Redfern, Manuel Montero-Odasso, Ervin Sejdić

**Affiliations:** Department of Biomedical Informatics, School of Medicine, University of Pittsburgh, Pittsburgh, PA 15261 USA.; Department of Physical Therapy, School of Health and Rehabilitation Sciences, University of Pittsburgh, Pittsburgh, PA 15261 USA.; Department of Computer and Information Sciences, Northumbria University, Newcastle upon Tyne NE1 8ST, U.K.; Department of Bioengineering, Swanson School of Engineering, University of Pittsburgh, Pittsburgh, PA 15261 USA.; Gait and Brain Laboratory, Parkwood Institute, London, ON N6C 5J1, Canada, and also with the Division of Geriatric Medicine, Department of Medicine, University of Western Ontario, London, ON N6A 3K7, Canada.; Department of Electrical and Computer Engineering, Swanson School of Engineering, University of Pittsburgh, Pittsburgh, PA 15261 USA, also with the Department of Bioengineering, Swanson School of Engineering, University of Pittsburgh, Pittsburgh, PA 15261 USA, also with the Department of Biomedical Informatics, School of Medicine, University of Pittsburgh, Pittsburgh, PA 15261 USA, and also with the Intelligent Systems Program, School of Computing and Information, University of Pittsburgh, Pittsburgh, PA 15261 USA.

**Keywords:** Walking, motor control, motor skill, movement control, lower trunk acceleration, wearables, gait, clinical informatics, machine learning

## Abstract

In adults 65 years or older, falls or other neuromotor dysfunctions are often framed as walking-related declines in motor skill; the frequent occurrence of such decline in walking-related motor skill motivates the need for an improved understanding of the motor skill of walking. Simple gait measurements, such as speed, do not provide adequate information about the quality of the body motion’s translation during walking. Gait measures from accelerometers can enrich measurements of walking and motor performance. This review article will categorize the aspects of the motor skill of walking and review how trunk-acceleration gait measures during walking can be mapped to motor skill aspects, satisfying a clinical need to understand how well accelerometer measures assess gait. We will clarify how to leverage more complicated acceleration measures to make accurate motor skill decline predictions, thus furthering fall research in older adults.

## I. Introduction

WALKING has been described as a skill that is acquired through motor learning [1]. The hallmark of a motor skill is a smooth and efficient movement that requires minimal attention [1]. Among older adults, the motor skill of walking varies widely [2]–[4] with declines in motor skill being among the most significant causes of falls [5], morbidity [6], and low quality of life [7]–[9]. Age-related decline in sensorimotor function further increases motor decline and may detrimentally change one’s gait [10].

Gait measures, such as gait speed, step length, and step temporal variability [7], [11], are used to characterize specific aspects of motor skill; however, these measures are somewhat limited. Some older adults may walk slowly with adapted optimal motor skill, while others may walk slowly with poor motor skill. Older adults with or without diagnosed disease may walk at clinically normal speeds with altered control [1], [12]. Other walking measures that are a better match to specific aspects of motor skill may prove to be useful when evaluating the gait of older adults.

The evaluation of the motor skill of walking considers multiple environmental factors. Evaluating walking in the clinic, while useful, is limited and may not capture the multiple dimensions of skills in everyday mobility. The recent emergence of wearable technology can capture numerous gait characteristics in various settings (e.g., clinical facilities, community settings, and in the home) [13]. Indeed, the amount of physical activity and human movement data collected from wearables is virtually unlimited; however, much of the data are not analyzed or used in a meaningful manner [14]. One way of making better use of this new data source is to develop metrics that match the motor skills of interest in older adults. This endeavor will require a collaborative effort between researchers in geriatrics of mobility and experts in engineering and data analytics.

One wearable technology that has gained prominence and has great potential to match with gait motor skill is accelerometry. Accelerometer assessment of gait is gaining clinical importance due to its simplicity and low cost. Acceleration gait measures (AGMs), derived or calculated from the raw values acquired with accelerometer wearables, capture body segments’ motion. Researchers have proposed that AGMs, particularly those derived from accelerations in the lower trunk, can be global indicators of the motor skill of walking [15]–[19]. AGMs are not only widely used [20] but can be proxies for center-of-mass dynamics [21], [22].

It is crucial to investigate motor skill in walking in relation to aging and illness. Trunk acceleration measurements have been used in the evaluation of normal aging [23], Parkinson’s disease [24], the impact of Alzheimer’s disease [25], and numerous other impacts on gait and balance [15], [26]. Previous studies found that older adults adopt more conservative gait patterns than younger adults, potentially to compensate for degeneration in physiological systems such as those associated with vision, sensation, and lower limb strength [23], [27]. These conservative gait patterns result in reduced walking velocities and accelerations, accompanied by reduced step length and increased step width [23].

**Fig. 1.**
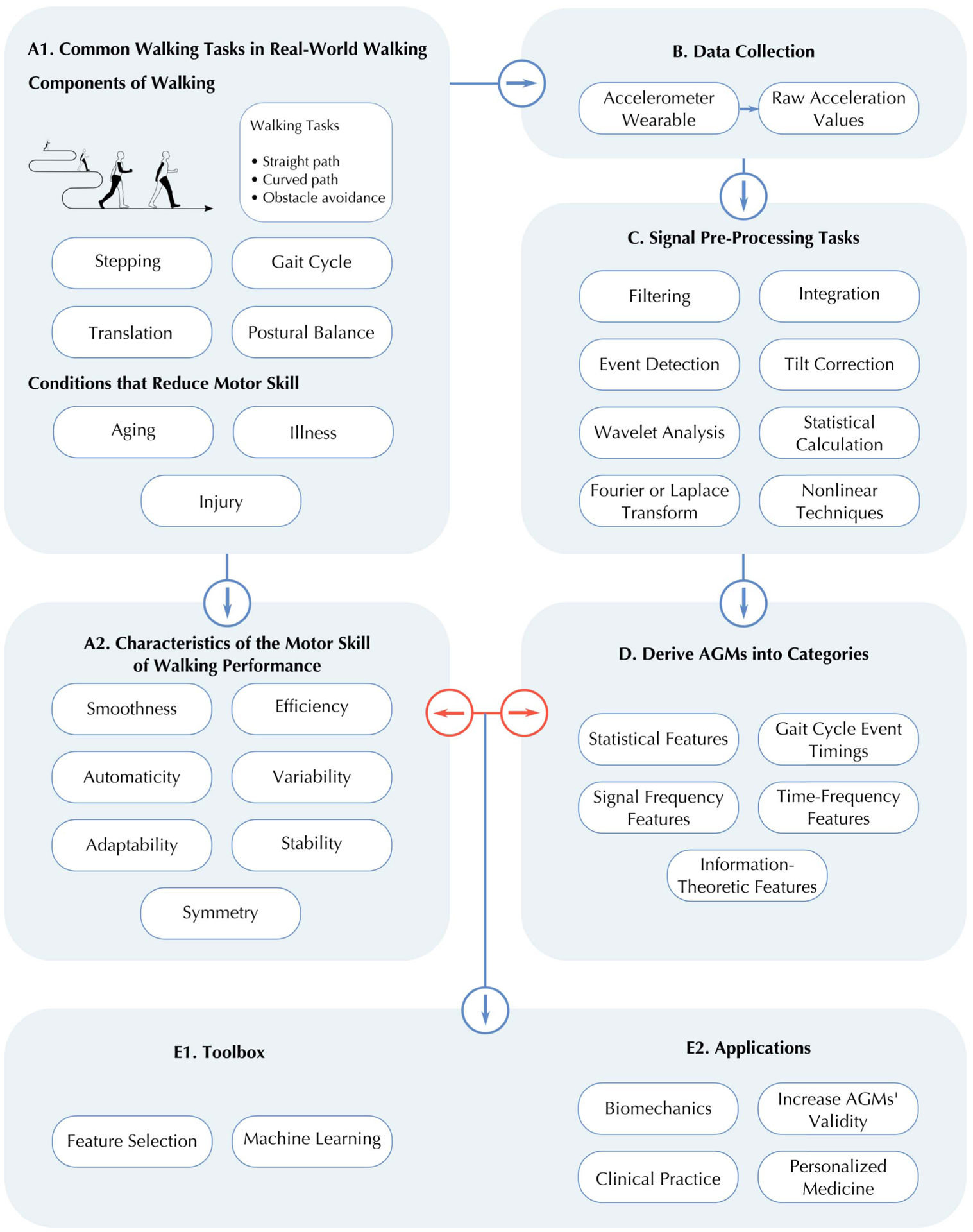
An overview of the pipeline mapping AGMs to motor skills. From top to bottom, (A1) defining common real-world walking tasks which can be impacted by normal aging, illness, or injury that are then mapped to (A2) seven characteristics of the motor skill of walking performance. (B) Accelerometer data collection results in raw acceleration values, which (C) undergo signal pre-processing before deriving AGMs. (D) These AGMs are grouped into categories that can then be matched to motor skills of walking. The red arrows show this review’s main contribution, where AGMs and motor skills can be mapped to each other. (E1 and E2) Subsequently, this mapping has various applications in clinical fields.

Mapping AGMs of the lower-trunk can help clinical gait interpretation by presenting quantitative gait variables stratified by domains (of the motor skill of walking) with clinical relevance [26]. To understand the motor skill in older adults’ walking, literature that combines the use of trunk-AGMs are reviewed. The structure of this review paper is divided into six areas, as summarized in Figure 1: motor skill and walking definitions (Figure 1–A1; Section II), accelerometer data collection (Figure 1–B; Section III), signal pre-processing tasks (Figure 1–C; Section III), deriving and categorizing AGMs (Figure 1–D; Section III), mapping the aspects of the motor skill of walking to trunk-AGMs (Figure 1–A2 and D; Section IV), and the applications and future directions of AGMs and motor skill in the clinical space (Figure 1–E1 and E2; Section V).

## II. Motor Skill of Walking

### A. Walking

Walking is defined as gait with intent, specifically, the control of the body’s center of mass and the continuation of movement; it involves multiple aspects of motor skill, which we call “the motor skill of walking” [1], [28]. Thus, walking is considered a form of “skilled movement,” which refers to a movement that “requires minimal attention to the individual components of the action, is goal-oriented, and learned through practice that proceeds through defined stages” [1], [29]. In the most general sense, walking can be thought of as moving the body through space by repetitive stepping (i.e., gait cycle) while maintaining postural stability and balance (Figure 1–A1) [30]. Postural stability refers to the inter-segmental coordination during locomotion, including the pelvic, torso, head control, and arm swing coordination. Balance is the ability to remain upright while walking. Thus, walking requires complex coordination to be successful [30].

The motor skill of walking is the set of learned coordinated actions that result in the body’s translation through space while maintaining postural control and balance [1], [28]. In various real-world environments (e.g., indoor, outdoor, crowded malls, uneven or littered ground), motor skill needs to be tractable. For example, this tractability can be defined for three general paths of walking: a straight path, a curved path, and an obstacle avoidance path (Figure A1) [1], [31]–[33]. In each case, changes in foot placement and postural adjustments are superimposed upon the gait cycle. Kinematic measurements during walking are used to quantify gait characteristics to evaluate the motor skill of walking. Several metrics can be calculated from these characteristics, which focus on the particular aspects of the motor skill of walking. Aligning the right metrics to the particular aspect of walking’s motor skill is imperative in defining healthy walking and impairments.

### B. Characteristics of Motor Skill

Motor skill, generally, refers to a motor task’s successful performance with consistency, efficiency, and the flexibility to adapt to different environmental constructs [34], [35]. The intact motor skill of walking produces a smooth and efficient translation of the body over the surface. A decline in motor skill often leads to coordination loss, haphazard timing of stepping, postural instability, and asymmetries in gait phases during walking. Each of these aspects of motor skill is important in evaluating locomotion towards defining impairments and guiding rehabilitation. Based on the literature search, we defined seven interrelated, critical characteristics of the performance outcome of the motor skill of walking:

- **Smoothness** is the consistent forward progression and regular, repeatable pattern of steps during walking [36]–[38]. Specifically, the smoothness of walking refers to the acceleration and deceleration of the trunk during walking. An interruption of the gait cycle events, such as heel strike and toe-off, can lead to uneven walking, characterized by an extended deceleration of the “the leading limb at heel strike and altered accelerations of the trunk to advance the trailing limb [1], [36], [37].”
- **Efficiency** is inversely related to the energy expenditure during walking; the higher the energy cost of walking, the lower the efficiency [1], [39].
- **Automaticity** is the reproducibility of walking motor skill with little attentional, central nervous system resources for guidance [1], [40].
- **Adaptability** is the set of accommodations to walking based on the response before or after the loss of postural balance (due to obstacles or biomechanical defects) [41].
- **Variability** (or regularity) is the change or fluctuation in walking from one stride to the next [42], [43]. Multiple metrics claim to measure gait variability, leading to many ambiguous definitions [13], [44]. While gait variability may include the discussion of stride-to-stride fluctuations [42], there are further definitions of variability, such as the change in other spatial parameters (e.g., foot clearance) and temporal parameters (e.g., duration of gait phases) from one gait cycle to the next [45].
- **Stability** in locomotion is a fundamental concept that relies on neural control given the system is mechanically unstable. Gait stability can be defined in multiple ways, from the simplest definition of the ability to walk without falling, to complex interactions of the neural controller with the mechanical system during the process of walking [41], [46], [47]. The latter includes concepts such as dynamic stability of the system [48]. In this review, we examine stability of walking by measuring variability in the temporal and spatial characteristics of the whole body and limbs. Please note that stability does not refer to dynamic/postural control, which is dependent on measures such as step width and step width variability [49], [50].
- **Symmetry** is the agreement between the actions and behavior of the lower limbs during walking [51], [52]. While smoothness and variability may include some aspects of symmetry, symmetry is more focused on the concordance of contralateral motion while walking [36], [53], [54].

The above characteristics can be evaluated in various locomotor tasks. For example, in straight-line walking, good motor skill is indicated by clinical measures of low gait variability (Figure A1). In contrast, for curved-path and obstacle-avoidance walking, good performance is indicated by clinical measures of high gait adaptability, particularly in step lengths and widths (Figure A1). Furthermore, in curved-path walking, a good motor skill can be indicated by high gait variability (Figure A1) [31]. Hallmarks of poor straight-path and curved-path walking are a decrease in walking speed, a decrease in stride length, a reduction in trunk movement, decreased strength and flexibility, and decreased balance (Figure A1) [55]. Signs of poor obstacle-avoidance walking are decreased swing velocity, rapid stepping to maintain balance, shorter step lengths, shorter obstacle-heel strike distance, and freezing/stopping in motion (Figure A1).

Motor skill is defined here as an intended voluntary task or goal-oriented motor action for walking [1]. The performance of these motor actions can be influenced by the environment or perturbations, but the response to these changes are not considered a part of the motor skill of walking [56]. For example, a gait perturbation such as a slip or trip in walking causes a response to regain stability and return to pre-planned locomotion where motor skills are engaged [57], [58]. Perturbations can be caused by cognitive, visual, mechanical (e.g., environmental) means, or pathological gait impairments [57], [59]. Perturbations do not refer to long-term changes in the system or environment, in which longer-term changes in one’s motor skill need to be made. Typically, one adapts to a perturbation by implementing faster, shorter, and wider steps [57]. Positive recovery from perturbations is related to increased stability and decreased variability of the motor skill of walking [57]. High variability as a response to a perturbation can indicate a risk for a future fall [27], [57]. However, perturbation studies, which often induce perturbations, are often risky for participants, especially older adults, and thus, there is little discussion of perturbations in this review.

The motor skill of walking is affected by age- and disease-related metabolic, cardiovascular, musculoskeletal, and neurological changes. Thus the altered motor skill of walking can be a functional indication of the aging system decline or subtle disease states. For example, for those who have Parkinson’s, walking in a straight path is more manageable than walking on a curved path or through/over obstacles [60]. Even in the presence of pain-free, adequate muscle strength and endurance, the difficulty in navigating curved-path walking and obstacle avoidance illustrate the disease-related altered basal ganglia to cortical communication impact on the timing coordination and adaptability of walking necessary for these walking tasks [61], [62].

## III. Acceleration Gait Measures (AGMs)

**Fig. 2.**
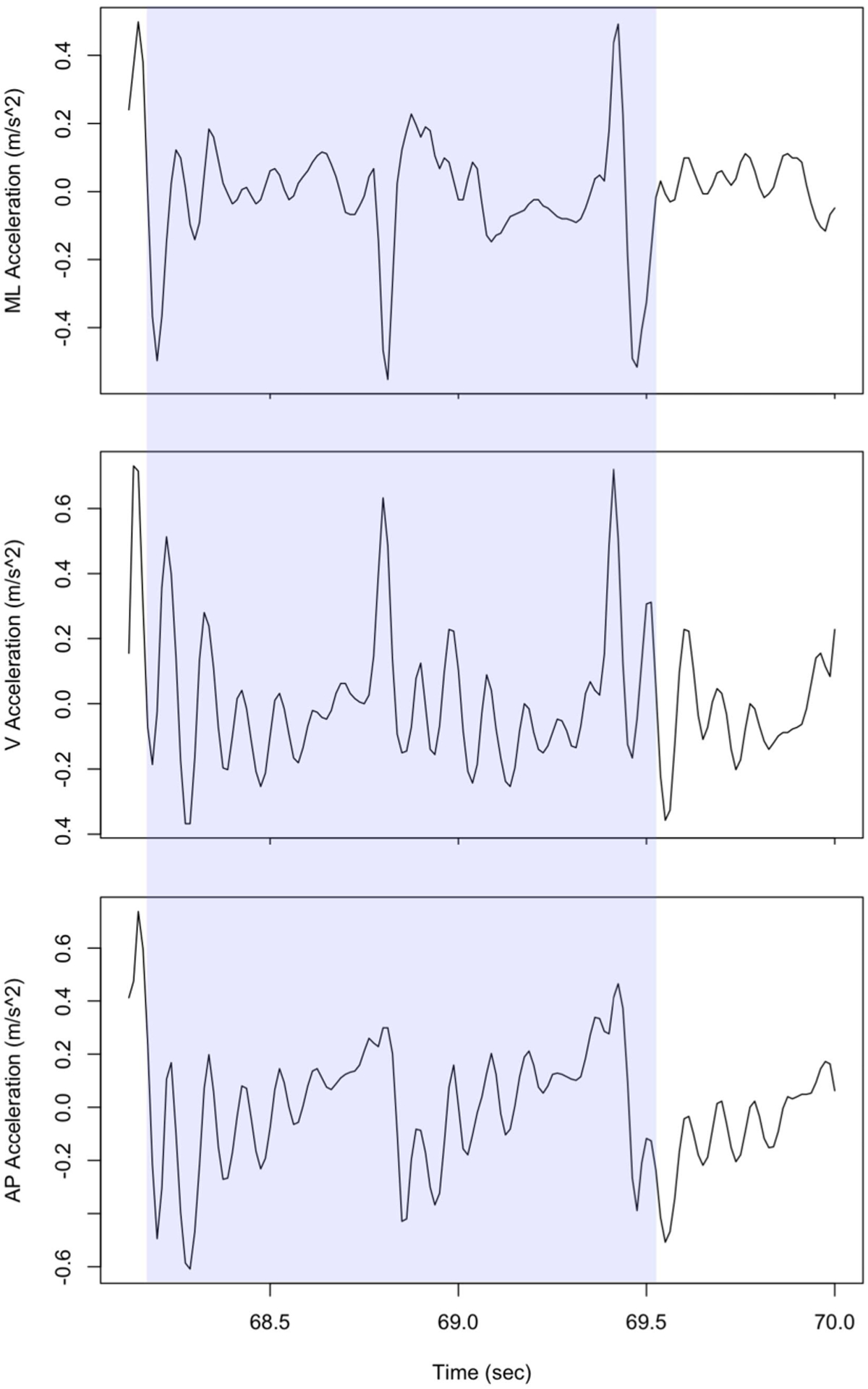
Example of acceleration signals (ML, AP, and V) from an accelerometer placed on the lower back. A full gait cycle of the right foot (starting from a heel strike) is shaded (data and gait extraction done by Dasgupta et al. [67]).

Accelerometers are used to study age- and illness-related changes in walking [63]. Accelerometers measure the accelerations of objects in motion along three orthogonal axes, often generally aligned with anatomical coordinates (e.g., mediolateral (ML), superior-inferior or vertical (V), and anterior-posterior (AP) [64]; these accelerations are time-series, and an example is shown in Figure 2. Inertial measurement units (IMUs) or wearable technologies that include an accelerometer component (e.g., fitness trackers) are preferred because the acceleration measurements can be used to validate the velocity of walking, distance walked, and the intensity of movement (Figure 1–B) [64], [65]. Since orientation is relative to gravity, accelerometers contribute to the identification of the objects’ rotation and orientation. These characteristics allow accelerometers to determine body postures [64].

In this review, we focus on accelerometer placement on the low-back region to approximate the body’s center of mass movement [66]. Research-grade accelerometers are often located at the level of the L3-L5 vertebrae and are most often used to measure spatial variability, smoothness, and symmetry of gait [36]. From a clinical perspective, low-back or lower-trunk placement succeeds because the trunk segment covers over half the body’s mass and is prioritized by the nervous system [17].

In this review, AGMs are grouped by the methodologies they are derived from 1) gait cycle event timings, 2) statistical features, 3) signal-frequency features, 4) time-frequency features, and 5) information-theoretic features (Figure 1–D). Examples of the AGMs for each category can be found in the Appendices (Section VIII).

The gait cycle is defined by the coordinated trajectories of each leg and each leg’s swing and stance phases during single support and double support [68]–[70]. Specific events of particular interest are heel contact, foot flat, heel off, mid-swing, and toe-off (see [71] for details on gait cycle parameterization) (Figure 2). Using AGMs to measure gait cycle characteristics often requires knowing these events and how often they occur (i.e., the number of strides). In the majority of studies, statistical summaries are performed on different gait cycle metrics over a time period [72]. Signal-frequency features are those acquired by the frequency spectra of the acceleration signals. Time-frequency features are features gathered through information from signal and time dimensions, using time-frequency functions [73], such as short-time Fourier transform and wavelet transformations. While some of the time-frequency features in this section may fit into the other AGM categories, they are specifically grouped here by how they are extracted from the acceleration signals. Information-theoretic features measure the amount of variability and uncertainty in the information context of a signal [16], [74]. Many of these features can be measured for each direction or a gait event (i.e., a stride).

In Table I, we define each of the categories and compare/contrast the differences between them. For the following attributes, we compare the strengths and weaknesses across AGM categories: 1) “Ease of calculation” refers to the difficulty of calculation of the AGMs, 2) “Directly applicable to clinical problems” refers to how contextually relevant the AGMs are without further explanation or back-calculation, 3) “Popular across literature” is how prevalent these set of AGMs are, 4) “Reduce complexity and dimensionality” is the extent to which AGMs capture a wide amount of information, and 5) “Tied to multiple aspects of walking” refers to how well the AGMs relate to walking elements (Table I).

## IV. AGMs in Action

### A. Motor Skill and AGMs

Understanding the use of AGMs as proxies for the aspects of the motor skill of walking will provide better clinical features for models that can potentially predict the motor skill of walking. Clinically, mapping motor skill characteristics (Section II-B) to categories of AGMs (Table I) may be capable of providing relevant and accurate measurements. In Table II, we summarized a selection of references for each of the aspects of motor skill–AGM mappings. By doing so, we also identify the existing gap by seeing how researchers have combined multiple features extracted from gait accelerometry signals into a derived AGM that could potentially be a marker for walking-related changes in physical function.

#### 1) Smoothness:

Walking smoothness is a high indicator of fall-risk in older adults. The most common way to measure smoothness is through root mean square [89]–[93], indices of harmonicity, or harmonic ratios (estimated for each of the three directions as the index of harmonicity) [36], [94]–[96]. Larger harmonic ratios can indicate a smoother gait pattern. In contrast, a lower ratio is found in older adults and older adults with unsteady gaits [16], [36], [80], [90], [93], [97]. During most modes of walking, the most significant impact on the harmonic ratio, due to increased age, is in the ML direction. Another way to measure smoothness is to measure the jerk-cost function from the gait movement [38], [98]. Lower jerk indicates higher smoothness in gait and higher motor control [38]. Power spectrum entropy of the acceleration signals can be used to differentiate persons likely to fall and persons not likely to fall, by their gait [105].

#### 2) Efficiency:

Efficiency, the inverse of energy expenditure, can also be used to assess the gait and evaluate balance in older adults [39], [127]. Energy expenditure was measured along with the center of mass accelerations in all forms of walking to come up with guidelines on how older adults can improve their walking [104]. Another way to measure efficiency is through measuring periodicity, precisely constant acceleration periods and changes [79], [86], [107]–[109]. While these AGMs are useful in measuring efficiency, validation methods such as measuring the oxygen rate during walking are often used [127], [128].

#### 3) Automaticity:

Automaticity often goes hand in hand with variability/regularity [40]. Many of the features that measure inter-step or inter-stride variability in walking can be indicative of automaticity. For instance, the coefficient of variation of stride velocity, coefficient of variations of the axial directions of accelerations, and swing time variability are measures of automaticity [1], [102], [103]. Other useful AGMs include the periodicity of accelerations [80], [90], [93], [99]–[101], and measures of efficiency [104]. For example, in patients who freeze or momentarily stop walking, a sign of Parkinson’s disease, these measures are particularly useful [129]–[132]. Moreover, automaticity becomes an important motor skill to investigate when studying cognitive impairment or load within aging adults [133].

#### 4) Adaptability:

Adaptability is a distinct aspect of the motor skill of walking, but it is very closely tied to the concepts of stability and variability/regularity. Adaptability is influenced by stability since people try to increase their stability in the ML direction to maintain an upright posture. Similarly, adaptability can be affected by variability/regularity, since people adapt back into their regular gait pattern when they are perturbed [120]. Statistical features of gait cycle events and the harmonic ratio can also be used to measure gait adaptability [16]. In obstacle avoidance studies [117]–[119], gait pattern adaptations were measured via step length variability. Step length variability is measured in the following studies: [90], [99], [100], [107], [108], [110]–[116]. The common measures of gait adaptability come from the use of Lyapunov exponents and entropy measures; while both variability and stability may use these measures, adaptability can be measured by examining the “continuum” of Lyapunov exponent and entropy values [134]–[136].

#### 5) Variability:

Typically, gait variability is calculated through simple measures (and by simple methods), such as step or stride length (or duration) [77]. Because accelerometers can collect massive amounts of data over time, they are especially useful in assessing stride-to-stride or step-to-step variability of walking [76]. Some common AGMs describing variability presented are:

- Standard deviation and coefficient of variation of the gait cycle events can directly measure variability [76].
- The median of the modal frequencies for the V, ML, and AP directions and the strength of the relative fluctuations in the phase progression can determine step/stride frequency [66].
- The autocorrelation coefficient of the signal can capture inter-stride variability [19], [76].
- The peak values of the first and second dominant periods of the autocorrelation function, simple statistical features, individual curve estimates, and adaptive peak thresholds can determine step/stride variability [43], [82], [83].
- Root mean square of the acceleration signal can be a measure of variability. For example, Rispens *et al.* define “movement intensity” as the root mean square of the acceleration [66], [79]–[81].
- Entropy, entropy rate, and Lyapunov exponents may be correlated with gait variability (as well as adaptability) [13], [16], [106], [137].

While many gait cycle events are used for variability, step duration is a much better measure than step length when investigating the loss of balance in older adults [23], [26], [75], [78]. Statistical summaries of step length, in conjunction with a low root mean square value, often indicate a typical gait pattern during walking. On the other hand, the autocorrelation coefficient of the signal and other signal-frequency features can better pick up characteristics of overall walking patterns. Finally, information-theoretic features can provide some insight into variability if other motor skill aspects are also being investigated [16]; for example, the regularity of a time series can be captured via entropy or entropic features [85].

Some specific examples in the literature have shown that measuring variability via AGMs is helpful to differentiate between classes of older adults. Older adults with neuromotor difficulties have one or more of the following: lower step/stride variability, lower step/stride frequency, and higher movement intensity in all forms of walking [23], [43]. Linear (mean velocity, the peak-to-peak amplitude of accelerations, root mean square, and frequency dispersion) and non-linear AGMs (Lyapunov exponent and entropy) can be used to measure the gait variability in patients with multiple sclerosis in lieu of simple footfall data [84]. Gait variability AGMs can be part of a clinical screening method for the locomotive syndrome since AGMs provide a complete, accurate, and personalized measurement of locomotive disorder in older patients with or without the musculoskeletal disease [138]. Gait irregularities and variability can also be measured to create a reference database, investigate outcomes in patients with gait disorders, and study rehabilitation for those with limited knee function [90], [99], [107], [108]. Similarly, other articles directly assess gait variability through trunk AGMs [91], [116], [139], [140].

#### 6) Stability:

To measure how people maintain gait stability, many researchers test a strategy of changing walking speeds or measuring accelerations. However, raw trunk acceleration data could enrich the measure of stability. Vertical accelerations can show the moments when toe-offs and heel strikes occur - decreased moments and low acceleration at heel contact, foot flat, mid-swing, and initial push-off are more prevalent in older adults [46], [81], [82]. High fractal values (from the maximum-likelihood-estimate analyses of accelerations) can indicate instability [27]. Additionally, measures such as root mean square [66], [79]–[81], standard deviations, and coefficient of variations of the acceleration signals can provide a better depiction of stability.

Non-linear aspects of stability can be described through dynamical systems analyses. Local dynamic stability is measured with the maximal Lyapunov exponent. Dynamical system analysis has been used to evaluate gait stability and falling risk [87]. A high local dynamic stability is indicative of good motor control and dynamically-stable gait. Another non-linear measure of stability are that has been used is the step stability index [43], [141]. The step stability index is a function of standard deviations of the intrinsic mode functions (derived from acceleration signals from the vertical direction) [43], [141]. The harmonic ratio, while it is often used to quantify smoothness or variability, can also be correlated with stability [142].

#### 7) Symmetry:

Similar to variability, fractal dynamics [76] and autocorrelation coefficient of the signal [76], the mean, standard deviation, coefficient of variation, and correlation of the gait cycle events [76], [79], [91], [111], [123], [124] are used to determine symmetry.

Symmetry can be derived from the autocorrelation function of the vertical acceleration signal [82], [101], [121]. There are more metrics of symmetry [51]: step asymmetry [122], symmetry ratio, symmetry index, gait asymmetry, and symmetry angle using step length, swing time, stance time, double support time, and an intra-limb ratio of swing time to stance time.

### B. Uses of Motor Skill–AGM Mapping for Gait-Related Outcomes

Mapping AGMs to motor skill can aid in differentiating gait-related outcomes through machine or statistical learning. In machine learning, there are two tasks: supervised learning and unsupervised learning. In the field of motor skill research, the goal of supervised learning is to learn a function from labeled data and approximate the relationship between the observable exposure and outcome variables in the data; in unsupervised learning, walking tasks, other gait-related, or motor decline outcomes are not labeled, and the goal is to deduce the relationships within the data.

Among the paradigms of classifiers for recognizing gait-related outcomes, regression, Naïve Bayes, support vector machines, decision trees, k-nearest neighbors, Hidden Markov Models, neural networks, and deep learning are the most popular. Typically, the pipeline for machine learning with acceleration signals follows the following steps: 1) pre-process the signals, 2) derive AGMs, 3) label the outcomes (if performing supervised learning), 4) use single or a combination of classifiers, and 5) applying models to test data to predict probabilities of class assignments.

However, with the use of machine learning and AGMs, it can be challenging to determine which selected features (AGMs) are less significant than others. Mechanistically, there are feature selection methods, such as forward or backward or recursive methods. However, it is more clinically useful to pick out relevant AGMs that fit the clinical problem’s context.

## V. Discussion and Future Directions

**Fig. 3.**
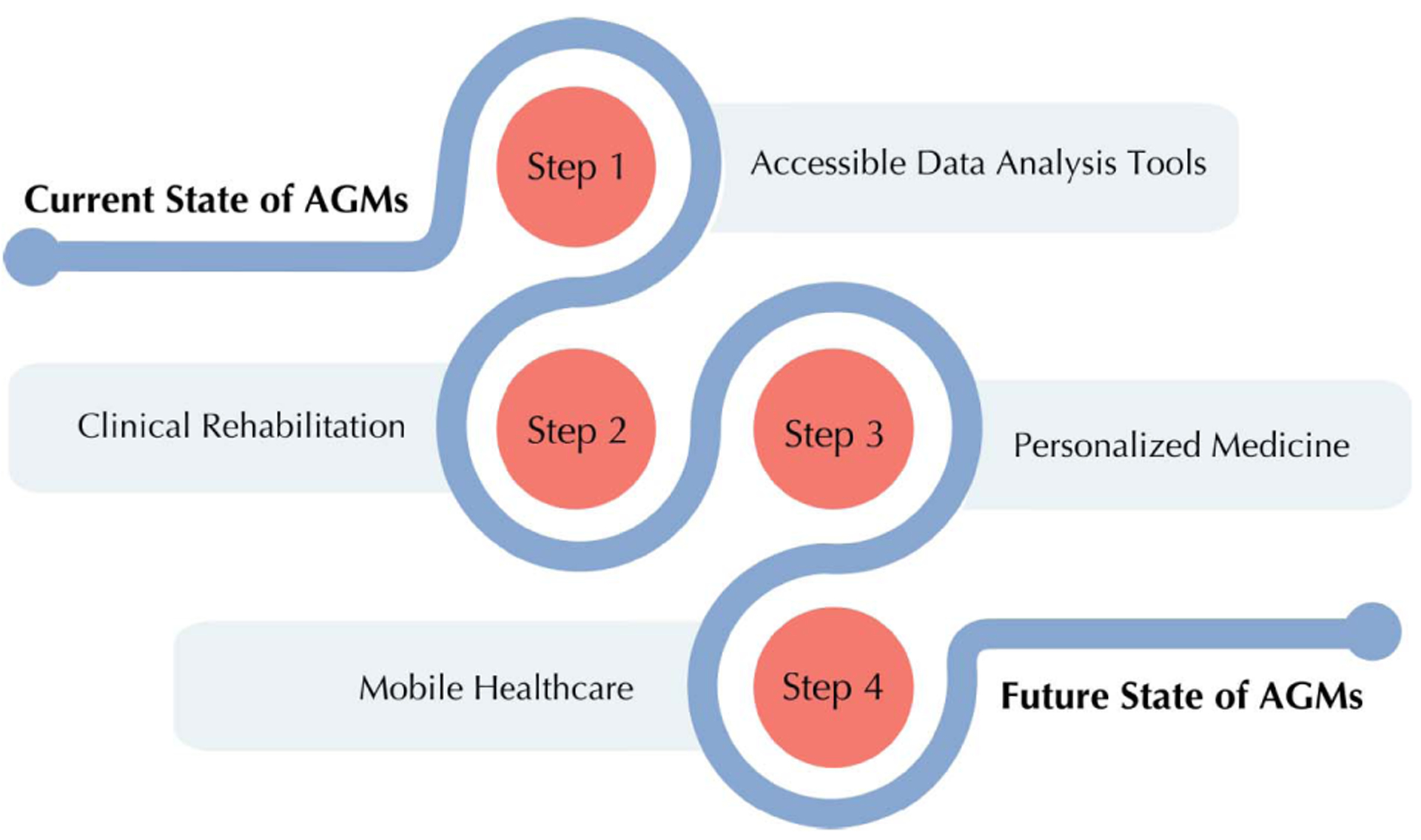
Comparison between the current and future state of AGM use in research.

The literature is overpopulated with multiple AGMs, and very few researchers can say they measure specific aspects of motor skill. For example, there appear to be several conceptual and data-driven clinical models that utilize AGMs for fall-risk assessment in various ways (Figures 3–4 from [13]). Thus, there are several issues to be addressed to move the field of gait and rehabilitation forward.

### A. Selection and Use of AGMs

Extracting AGMs from raw acceleration values is a natural step in biomedical informatics research. With the increased use of artificial intelligence, feature selection and specification are necessary for scientists to build statistical models to make predictions in the context of their problem. Clinical researchers in rehabilitation and physical-activity sciences may find utility and insight from conducting more studies in observational and clinical trials with AGMs to further the field.

However, the current selection and use of AGMs in research have limited value because of a lack of gold-standard information from acceleration measurements. Only a few studies have compared various AGMs within the same sample or dataset, let alone in different study designs. Moreover, there is a discrepancy in how AGMs are used between age, sex, gender, and disease groups. Further, previous research is limited to comparing AGMs to common simple gait measurements [143]. Collectively, research has a minimal consensus on the validity of using many of these AGMs.

There is little consensus on the most useful AGMs for analyzing locomotion in general, particularly with an accelerometer located on the lower back. There are very few studies that examine more than one AGM from one dataset [144]. Most of the current single AGMs studies only differentiate generalized populations (e.g., older adults vs. young adults) as opposed to more specific groups (e.g., older adults who are more prone to falling vs. older non-fallers). To improve the accuracy of the AGMs for detection of gait impairment, future researchers need to combine multiple AGMs through modeling [144]. Analyzing AGMS collected pre- and post-intervention can examine discriminative ability, responsiveness and construct validity for various AGMs [144], [145].

### B. Contribution of AGMs to Gait & Motor Skill Research

The contribution potential of a critical analysis of AGMs and the aspects of the motor skill to which they are mapped is substantial. As iterated in the introduction, gait impairments and “poor motor” skill of walking are observed across various morbidities. These gait impairments can have significant consequences on the quality of life of individuals. In the clinical space, gait and the motor skill of walking is often evaluated using observational scales and performance-based tests, such as the Timed Up and Go test. This evaluation can only be done by trained health professionals and may not prevent future gait-related incidents, such as falls. However, the addition of accelerometers and AGMs can provide a more continuous assessment of a person’s gait and walking skill. For example, Salarian *et al.* developed a Timed Up and Go test using from five to seven accelerometer sensors; which had good psychometric properties at a pilot study for Parkinson’s patients; main features that demonstrated association with the Unified Parkinson’s disease rating scale, extracted from instrumented Timed Up and Go are step counting, seconds, peak arm velocity, cadence, stride and turning and among the sub-elements of the instrumented Timed Up and Go test, gait, turning, and turn-to-sit were the most reliable [146].

### C. Issues in Validity and Interpretation of AGMs

There are multiple construct validity issues with the use of AGMs, because of the various methods for the derivation of an AGM from gait accelerometry and no known means to compare across the derived AGMs. It is not certain if various AGMs represent the same findings of the motor skill of walking, or if differences in the ability of various AGMs to distinguish the level of physical functioning in daily life.

In the studies that we have identified that investigate the impact of aging and illness on specific walking tasks, older adults adopt more conservative and compensatory gait patterns [27]. Older adults typically have reduced walking velocity and trunk-accelerations accompanied by reduced step length; these reduced accelerations are possibly induced to compensate for degeneration in vision, sensation, and lower-limb strength [23]. Notably, in straight path walking and curved-path walking, older adults have increased sub-movements, deceleration, and hesitancy [38].

Furthermore, few studies have researched how multiple AGMs within the same sample can effectively improve a statistical model. Several investigators report individually defined indexes of the acceleration signal, derived by proprietary algorithm methods [147], [148]. Little replication of AGMs in the same target population exists, including by the same investigator in subsequent studies of a similar sample. As a result, the clinical investigator has little to base an informed decision or intervention about the usefulness of derived AGMs to describe, detect, and monitor walking abnormalities. Therefore, there is an obligation for further study into comparing AGMs in a more standardized way.

### D. Addressing Barriers to Future Use

Without reliable and accessible tools within an established signal pre-processing pipeline, the use of AGMs in research cannot be feasible. Acceleration signal pre-processing can be a time-consuming task and can get in the way of diagnosing or analyzing a clinical problem. The assessment of gait in the clinical space lacks maturity with the use of these signal pre-processing tasks.

This paper does not address the deeper issues of data collection or signal pre-processing. Data collection involves technical issues [149], such as sampling rates used, frequency response requirements for different tasks, placement and alignment of the accelerometer on the trunk [26], and how they are attached for long-term and short-term use. To derive AGMs, there are several pre-processing steps that can be used to prepare the signal data [86], [150], such as filtering or extracting noise from the signals [151]–[153], event detection and labeling [66], [71], [154]–[156], wavelet analysis and decomposition [68], [157], [158], Fourier or Laplace transformations [159], integration [150], [160], [161], tilt correction [86], nonlinear techniques [158], statistical calculations [67], [162]. A non-exhaustive list of signal pre-processing tasks can be found in Figure 1–C.

Computing languages, packages, and toolboxes will come and go, but there will always be a constant need for technological tools that are more accessible to researchers of all levels. Some of the attributes any tool processing the acceleration signal to AGMs should have are the ability to visualize accelerations, packages that can filter out signal noise, and the ability to extract signal features into a data structure that can later be used in statistical modeling. While MATLAB, Python, and the other current tools have all of these pieces, tools with greater ease of use and reduced programming requirements could make these measures more available to a broader audience of researchers and clinicians.

### E. Future State of AGM Use

In Figure 3, the future of this field and how gait accelerometry research can be ameliorated through the use of AGMs, not just in the clinical space but also in the hands of patients and consumers. For instance, AGMs combined with electronic health and medical records may be used to identify those with a high risk of falls [163]. Since wearables are increasingly reducing in size, they can be used as a means to provide digital medicine with a harmonious set of biomarkers (risk, diagnostic, monitoring, prognostic, etc.) [164].

## VI. Conclusion

The use of AGMs is increasing due to the ease of use and low cost. The ultimate goal is to develop screening measures for a walking-related physical-function decline. Also, AGMs could inform intervention strategy and monitor outcomes. However, currently, there is a disparity in the literature reviewing the different mapping of AGMs to aspects of motor skill. In this review, we characterized the three different modes of walking, defined seven motor skill aspects of walking, categorized five broad categories of AGMs, and discussed the typical AGMs used for the aspects of the motor skill of walking. This review will elucidate how AGMs supplement simple measures and improve our understanding of how AGMs can be used to investigate locomotion. Linking motor skills of walking to AGM metrics will prove useful in quantifying declines due to aging and other neuromotor factors. In application, AGMs have been used to detect differences and changes in motor performance due to learning/expertise, or task and environment manipulations. In conclusion, AGMs are a promising component of motor skill research, which can help older adults’ quality of life and reduce the strain on healthcare.

## Supplementary Material

supp1-3044260

## Figures and Tables

**TABLE I:** Qualitative Attributes of the Different Categories of Acceleration Gait Measures

Attributes	Categories of AGMs				
	Gait Cycle Event Timings	Statistical Features	Signal Frequency Features	Time-Frequency Features	Information-Theoretic Features
Ease of Calculation	○	●	○	○	○
Directly Applicable to Clinical Problems	●	●	○	○	○
Popular Across Literature	●	●	●	○	○
Reduce Complexity and Dimensionality	○	●	●	●	●
Tied to Multiple Aspects of Walking	●	●	●	●	●

● = strength

○ = weakness

**TABLE II:** Literature Citations That Depict the Mapping Between the Seven Aspects of the Motor Skill of Walking and Acceleration Gait Measures

Aspects of Motor Skill	Categories of AGMs				
	Gait Cycle Event Timings	Statistical Features	Signal-Frequency Features	Time-Frequency Features	Information-Theoretic Features
Variability	[23], [26], [75]–[78]	[16], [19], [66], [71], [76], [79]–[81]	[16], [43], [82]–[84]	[16], [85]	[16], [84], [85]
Stability	[46], [81], [82]	[66], [79]–[81]	[86]	-	[87]
Smoothness	[88]	[89]–[93]	[16], [36], [80], [90], [93], [93]–[97]	[38], [98]	-
Automaticity	[1], [80], [90], [93], [99]–[104]	[1], [79], [80], [101], [104], [105]	[80], [90], [93], [99]–[101]	-	[106]
Efficiency	[79], [86], [107]–[109]	-	[79], [86], [107]–[109]	-	-
Adaptability	[90], [99], [100], [107], [108], [110]–[120]	-	[16]	-	-
Symmetry	[18], [51], [76], [79], [82], [91], [101], [111], [121]–[124]	[16], [76], [79], [82], [86], [91], [101], [121], [124], [125]	-	-	[76], [126]
