# Supplementary material for: Acceleration Gait Measures as Proxies for Motor Skill of Walking: A Narrative Review
Source: IEEE Trans Neural Syst Rehabil Eng. Author manuscript; Available in PMC 2021 Mar 26. (PMC7995554; doi:10.1109/TNSRE.2020.3044260)

Appendices

9 Supplemental Information on Acceleration Gait Measures

9.1 Gait Cycle Event Timings

Combining the knowledge of the gait cycle with simple measures, such as gait speed and speed variability [165], we can estimate the times of each of these events and phases (e.g., lengths and times of stance, swing, step, and strides [90,99,100,107,108,110–116]). Cadence [80,97,107,108,112,114, 116,121,166,167], stride frequency [79,82,109,122], step or stride irregularity [168], stride or step amplitude [169], and stride frequency variability [66,156] can be calculated with AGMs. Other articles may provide some good insight into how gait cycle AGMs are measured and used [139,170–173].

9.2 Statistical Features

- The mean trends of the acceleration signal amplitudes in the ML, V, and AP directions from different gait phases and activities can be used to characterize a feature of the specific gait phase or activity [67,174].
- Correlation and covariance of the acceleration signal amplitudes between the pairs of ML, V, and AP directions (ML-V, ML-AP, V-AP) can help elucidate differences among activities that involve translation in just one dimension [67]. For example, in Dasgupta et al. and Sejdic et al., correlations and covariances were calculated as a basic statistical features [16,67]. Additionally, the auto-covariance can also be used to estimate stride time and stride regularity [66,71]. Cross-correlations are a measure of similarity between signals [175]. The autocorrelation coefficient of the acceleration signal is the cross-correlations of a signal with itself [71,83].
- The coefficient of variations of gait parameters, like gait speed and stride time, can help us see the fluctuations between strides, steps, or other gait phases [42].
- Skewness and kurtosis of the acceleration signal amplitude in the ML, V, and AP directions respectively describe the lack of symmetry and whether the amplitude signals are peaked or flat relative to a normal distribution [16,86,125].

- The maximum and minimum acceleration signal amplitude in the ML, V, and AP directions can be used to estimate stride time variability [66].

- Mean, standard deviations, median, percentiles, correlation, covariance, coefficient of variation, skewness, and kurtosis of variables and gait events/phases, which are not raw acceleration signals, can also be done to reduce the complexity of those features [66].

- The root mean square of the acceleration signal amplitudes can measure the magnitude of the acceleration signal and can be correlated with walking speed [89, 95, 96, 176] and can be used to estimate the variability of the signal [37, 177].

9.3 Signal-Frequency Features

- The peak frequency is the frequency when the maximum spectral power takes place, and it can be an indicator of gait cycle events [16,86,178–180]. The spectral centroid, like peak frequency, is another marker of spectral change in signal [16, 86, 178].

- The bandwidth of the signal expresses the spectral spread of the signal and is often used to differentiate between walking tasks [16, 86].

- The index of harmonicity is the spectral power of the basic harmonic divided by the sum of the power of the first six harmonics [181, 182]. Harmonic ratios can evaluate the harmonic balance and content for a stride, step, or gait phase [16, 94]. The total harmonic distortion of the signal is represented by how much distortion the signal-frequency has from other factors [183]. All these measures are known to assess the motor skill of gait [184].

9.4 Time-Frequency Features

- Estimation of initial contact/final contact of the foot can be done with wavelet transformations, bi-orthogonal spine wavelet, and jerk cost functions [98, 185].

- Wavelet transforms can be used to determine the relative energy from each time-frequency band [16]. Overall, wavelet transforms of acceleration signal data are useful in segmenting gait cycle events, gait surveillance and monitoring, and many advanced statistical calculations such

as principal components analysis and independent components analysis are used in conjunction with wavelet transformation [144].

- Wavelet entropy measures the "degree of time-frequency based order-disorder of the acceleration signal," and it is computed by wavelet decomposition [16].

9.5 Information-Theoretic Features

- Entropy measures, such as multi-scale or Shannon entropy, can measure the uncertainty of a specific variable, which means that the entropy rate can be used to measure the regularity of the acceleration signal [13, 16]. Regarding gait, it can be used to assess the predictability of acceleration signals (from one step/stride to the next step/stride) [106]. These entropy measures can be derived from the recurrence quantification analysis. Recurrent quantification analysis quantifies "deterministic structures and non-stationarity" [186].

- Cross-entropy rate measures the entropy rate between data points from two different signals [16]. Cross-entropy between acceleration signals was used to differentiate walkers with Parkinson's disease without Parkinson's disease [187].

- Lempel–Ziv complexity can be used to assess discrete-time signals in terms of their dynamics, complexity, bandwidth, and variability [126]. Lempel-Ziv complexity has been used as a measure of complexity in the non-linear analysis of gait patterns in those with Parkinson's disease [188].

- Fractal dynamics can capture the non-linear aspects of gait, typically through analysis like the Detrended Fluctuation Analysis [75, 189]. Hausdorff et al. studied stride-to-stride changes using fractal dynamics [136].

- Lyapunov exponents can measure disruptions or perturbations in signals, which can expose new insights about gait variability [189–192]. For example, local dynamic stability can be measured by evaluating the maximal finite time Lyapunov exponents [190,193]. Local dynamic stability is used to measure the gait stability and quality [66].

- Newer phase-dependent Lyapunov exponents and entropy measures are used to improve fall-risk prediction. For example, phase-dependent

Figure A1: For each of the three walking tasks: straight-path, curved-path, and obstacle avoidance, we define "good" motor skill of walking.

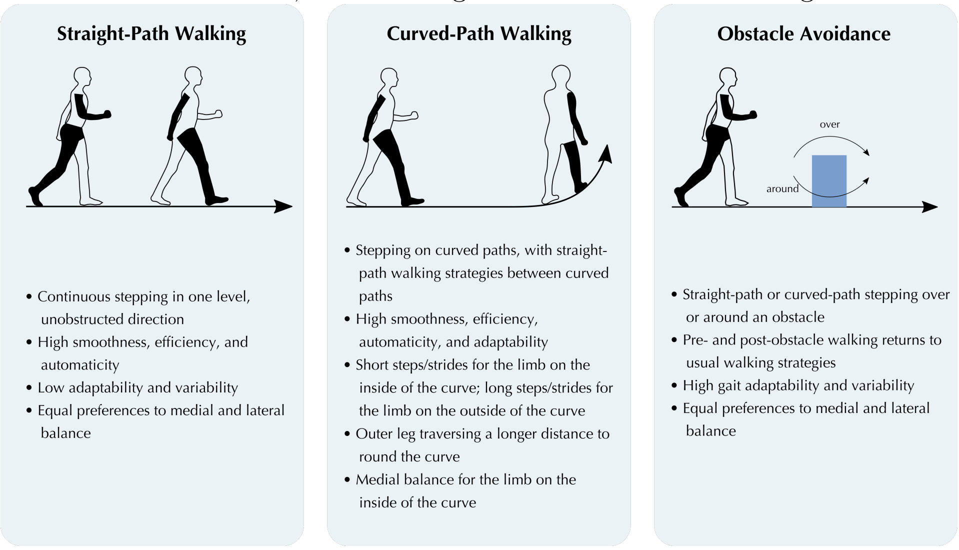

generalized multiscale entropy was used to differentiate between fallers and non-fallers; this type of analysis can capture non-linearity and involves both spectral decomposition analysis and phase-dependent analysis [194]. Whereas, phase-dependent Lyapunov exponents can be used to analyse local dynamic stability, such which cueing strategies affect gait stability in different gait phases [195].

- Orbital stability measures, such as Floquet multipliers, "quantify how purely periodic systems respond to perturbations discretely from one cycle to the next" [196–199].

10 Supplemental Figures

Figure A2: "Gait Cycle Event Timings" AGMs that measure aspects of motor skill. All these AGMs, encapsulated by an encompassing box, are correlated with each of the aspects of the motor skill.

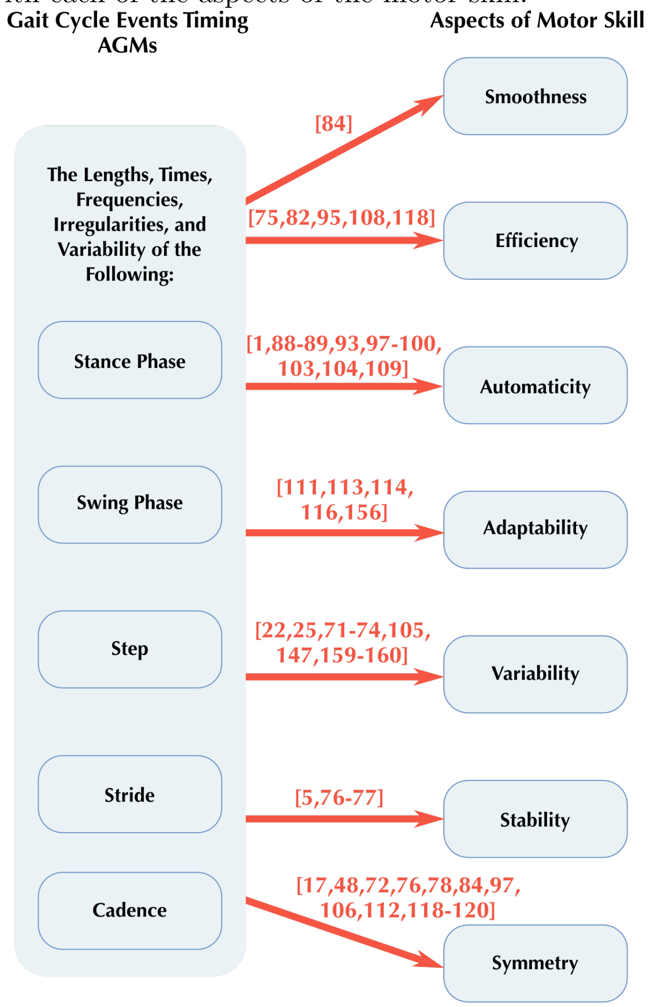

Figure A3: "Statistical Features" AGMs that measure aspects of motor skill.

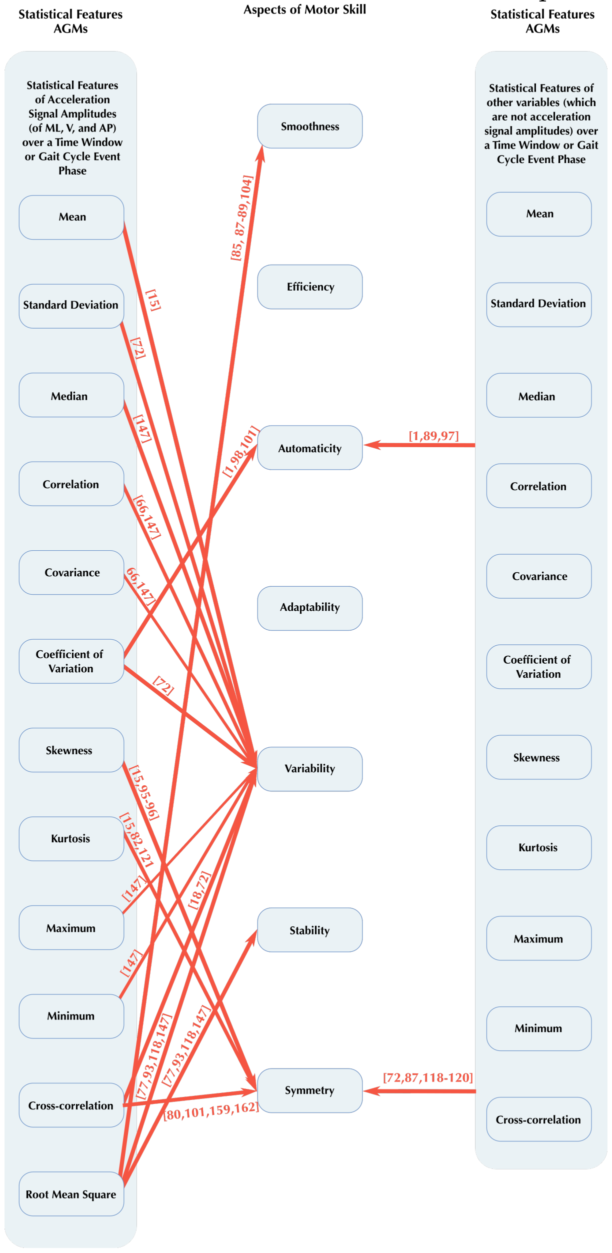

Figure A4: “Signal Frequency Features” AGMs that measure aspects of motor skill.

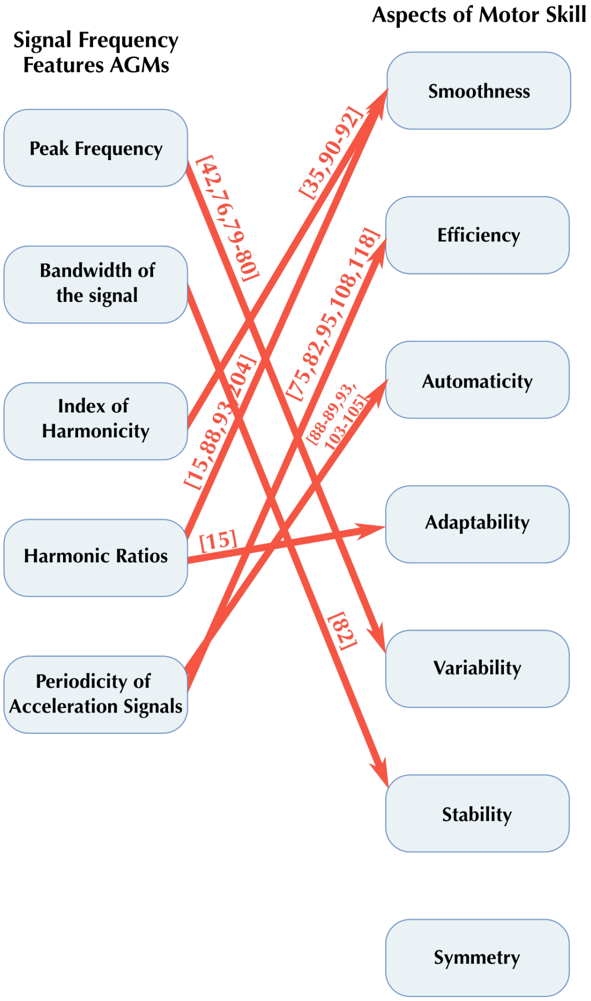

Figure A5: "Time-Frequency Features" AGMs that measure aspects of motor skill.

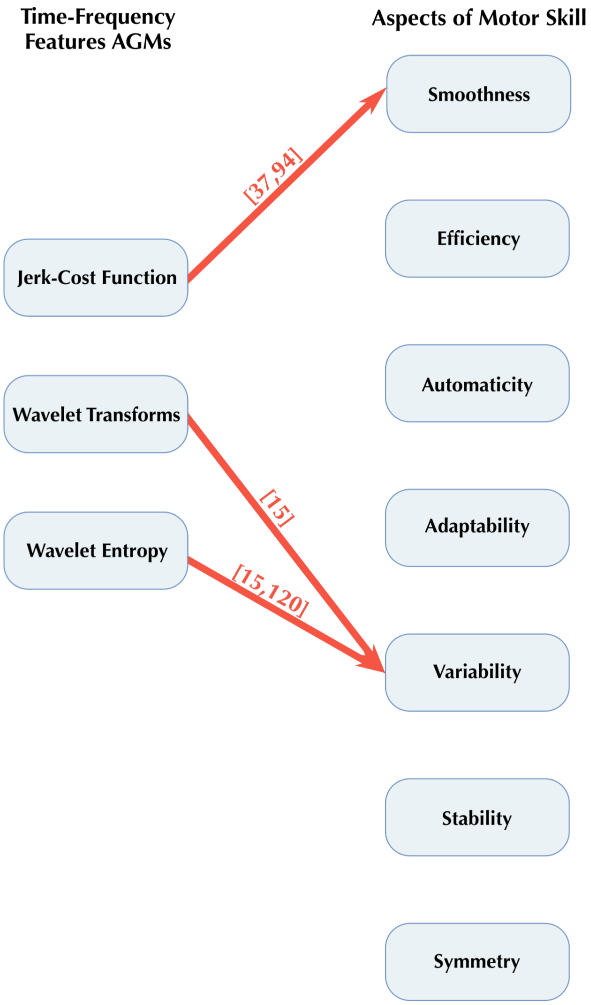

Figure A6: "Information-Theoretic Features" AGMs that measure aspects of motor skill.

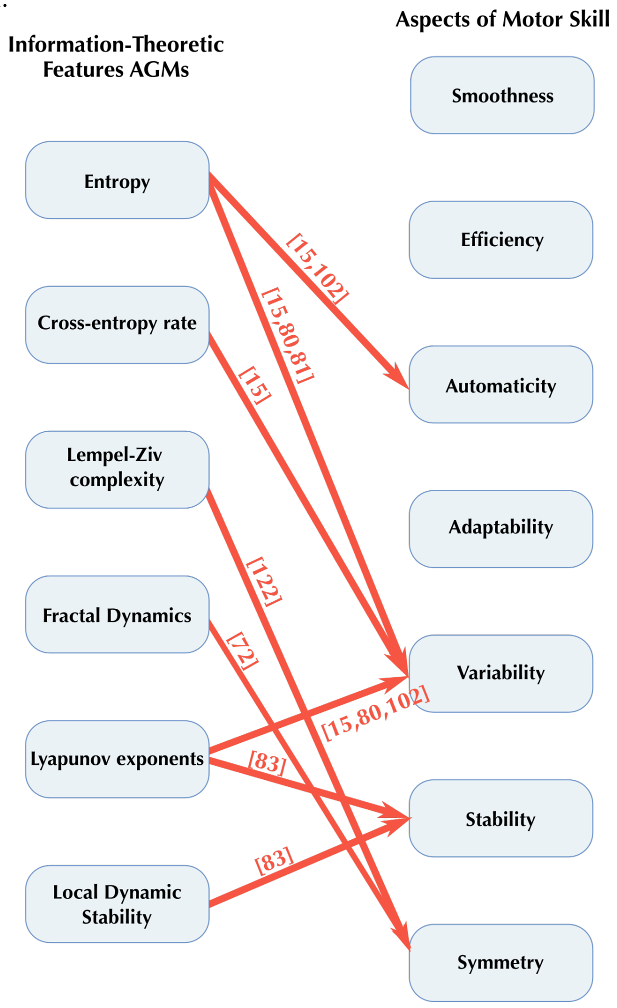

Supplement: supp1-3044260 [file NIHMS1678828-supplement-supp1-3044260.pdf]
